# The Impact of Cost of Living on the Quality of Life of Cystic Fibrosis Patients: A Study in Greece

**DOI:** 10.7759/cureus.63009

**Published:** 2024-06-24

**Authors:** Dimitris Dainavas, Pantelis Stergiannis, Panagiota Manthou, Georgios Lioliousis, Filia Diamantea, Pavlos Myrianthefs, Georgios Fildissis

**Affiliations:** 1 Department of Nursing, School of Health Sciences, National and Kapodistrian University of Athens, Athens, GRC; 2 Critical Care Medicine, University of West Attica, Athens, GRC; 3 Intensive Care Unit, 1st Department of Respiratory Medicine, Thoracic Diseases General Hospital Sotiria, National and Kapodistrian University of Athens, Athens, GRC; 4 Adult Cystic Fibrosis (CF) Unit, Sismanoglio Hospital, Athens, GRC

**Keywords:** general health questionnaire, cystic fibrosis (cf), physical functioning, quality of life (qol), cost of living

## Abstract

Introduction

Cystic fibrosis (CF) leads to the impairment of multiple essential organs and systems in the human body. The objective of this study was to analyze the financial consequences of having cystic fibrosis (CF) on patients, evaluate their general state of health, and specifically investigate the impact of living expenses on their quality of life.

Methods

The data were collected using three tools and a form for personal information entry. The first questionnaire was employed to quantify fluctuations in patients' cost of living. The Greek variant of the Short Form Questionnaire-36 (SF-36) and the Greek version of the Cystic Fibrosis Quality of Life (CFQoL) were implemented to assess quality of life. Statistical significance was set at p < 0.05, and analyses were conducted using SPSS statistical software.

Results

The study obtained a response rate of 93.2%, with 105 participants consenting to and effectively finishing the questionnaire. The mean age of the patients was 32.1 years, with 46.7% being female and 53.3% being male. Medication was being administered to 46.7% of the patients. The condition incurred an average cost of 767€ in the preceding semester. The maximum cost was 1007€. Patients with a higher monthly family income and those who were taking medication exhibited superior physical performance and functional capacity.

Conclusion

The research emphasizes that implementing causative treatment and minimizing hospitalizations can potentially enhance life satisfaction. The findings suggest possible approaches to enhance the quality of life in people with cystic fibrosis, in conjunction with the implementation of novel or enhanced treatment modalities.

## Introduction

Cystic fibrosis (CF) is caused by a gene mutation on the 7th chromosome, resulting in the dysfunction of several vital organs and systems in the human body [1]. The disease's primary symptom is the development of extremely viscous secretions in a number of the body's organs, leading to the slow destruction of vital organs and the eventual collapse of the body's systems. It is the most common genetic disorder that impacts the White population worldwide [2].

Health-related quality of existence (HR-QoL) refers to several dimensions of an individual's daily life, such as overall assessment of their physical, psychological, and social health, as well as their degree of functioning and well-being. Assessing HR-QoL can be difficult since it depends on the individual's subjective evaluation of their circumstances, as well as their beliefs and views. Health-related quality of life encompasses an individual's subjective assessment of their social standing, which includes their own values, goals, and the expectations they have from society [3].

Several researchers have assessed the quality of life (QoL) of individuals with CF in relation to psychological, demographic, and clinical factors [4-8]. Multiple methodologies have been devised in various programming languages with the explicit purpose of evaluating the standard of living, and a multitude of these methodologies have been utilized in medical research studies [9-13]. Recent studies have demonstrated the utilization of QoL as a measurement to assess the efficacy of clinical trials in both adult and paediatric populations [14, 15]. The assessment was undertaken in combination with antibiotic therapy and after lung transplantation, as stated by researchers [16, 17]. The literature discusses several facets of QoL dimensions, including the management of medical concerns and adherence. Importantly, there is a limited connection between quality of life ratings and many measurements, including lung function, body mass index, and exercise capacity, which are commonly used to assess the seriousness of a disease [6-8]. The predominant focus of research in the global literature for CF patients is entirely on evaluating their quality of life. Only a limited number of researches have investigated the recording of illness expenses [18-21].

This study aimed to examine the financial implications of living with cystic fibrosis (CF) on patients, assess their overall well-being, and specifically explore how the cost of living affects their quality of life.

## Materials and methods

The ethics committee of the Department of Nursing of the National Kapodistrian University of Athens granted ethical approval for this study (322/10-06-2020), and after written informed consent, data from 105 consecutive patients admitted to ICU between November 2020 and December 2023 were collected.

Inclusion and exclusion criteria

The proposed study was carried out in a General Hospital of Attica and specifically at the Adult Cystic Fibrosis Unit, where patients with CF are followed. The exclusion criteria of the participants in the survey were as follows: (1) Patients who refused consent, (2) patients who were unable to give consent, (3) patients <18 years old, and (4) patients who could not speak the Greek language.

Study design

The data was collected using three tools and a form for personal information entry. The initial questionnaire is employed to quantify fluctuations in patients' cost of living. The following questions are open-ended and pertain to the demographic characteristics of the sample, disease-related expenditures, income loss due to job changes, and other expenses necessary to manage the disease's consequences. The questionnaire was developed by Stergiannis et al., and permission was obtained for its utilization [22]. Two instruments were implemented to assess the quality of life. At first, we implemented the Greek variant of the Short Form Questionnaire-36 (SF-36). This instrument was developed to evaluate the quality of life of both patients and the general public, and it is entirely free to use. It includes inquiries regarding the individual's social function, vitality, general health, discomfort, physical function, and role. Permission for its use was also obtained. Additionally, it comprises inquiries regarding the significance of emotions and mental health [23].

The second measure used to assess the target population's quality of life is the Greek version of the Cystic Fibrosis Quality of Life (CFQoL), which was created and licensed for the use of CF patients. It includes questions regarding the individual's physical function and mobility, social life, therapy, symptoms, emotional functioning, future worries, interpersonal relationships, body image, and career [24]. Participants completed both questionnaires to capture the cost of living and its impact on quality.

Statistical analysis

After data collection, statistical analysis was performed, which included, in addition to the descriptive results, the selected comparisons in order to assess both the cost of living of CF patients and their quality of life. In the case of quantitative variables the mean, standard deviation, median and interquartile range were calculated, while in the case of categorical variables, the absolute and relative frequencies were calculated. The normality test of the quantitative variables was performed by the Kolmogorov-Smirnov test. Student's t-test was used to compare means between two groups, whereas ANOVA was used to compare means of three or more groups. Pearson's correlation coefficient was used to investigate the existence of a correlation between two quantitative variables following the normal distribution. Spearman's correlation coefficient was used to investigate the existence of a correlation between two quantitative variables that do not follow a normal distribution or between ordinal variables. In case the dependent variable is quantitative and more than two statistically significant variables are obtained in the bivariate analysis, the multiple linear regression model was used. All reported p-values are two-sided values. Statistical significance was set at p < 0.05, and analyses were conducted using SPSS statistical software (IBM Corp., Armonk, NY, USA).

## Results

The study achieved a response rate of 105 (93.2%) participants agreeing to and successfully completing the questionnaire. The demographic attributes of these patients are displayed in Table 1. The average age of the patients was 32.1 years, with 49 (46.7%) being female and 56 (53.3%) being male. In addition, 29 (27.6%) patients were in a marital relationship (Τable 1). The majority of patients (n=58, 55.2%) were permanently residing in Attica, and 47 (44.8%) patients were permanently residing outside Attica. Additionally, it appeared that the residents of Attica had superior emotional functioning. In terms of educational attainment, 3 (2.9%) individuals had completed primary school, 10 (9.5%) had completed high school, 20 (19%) had completed vocational school, and 72 (68.5%) had obtained a higher education degree. Almost half of the patients (n=49, 46.7%) were being administered medication.

**Table 1:** Demographic Data α mean, standard deviation

Characteristics	Ν	%
Gender		
Male	56	53.3
Female	49	46.7
Age^α^	32.1	10.4
Residence		
Out of Attica	47	44.8
Attica	58	55.2
Marital Status		
Married	29	27.6
Single/ divorced/ widowed	76	72.4
Number of Children		
0	85	81
1	13	12.4
2	5	4.8
3	1	1
4	1	1
Number of persons living together		
0	15	14.3
1	30	28.6
2	26	24.8
3	24	22.9
4	8	7.6
5	2	1.9

The descriptive results for the SF-36 scales are presented in Table 2. Higher SF-36 scores also indicate better quality of life. Scores on the mental and physical health summary scales were lower than 50 indicating that patients' quality of life was worse than average for both mental health and physical health (Table 2).

**Table 2:** The descriptive results for the SF-36 scales.

Scale	Mean	Standard deviation	Diameter	Minimum Value	Maximum Value
Physical functionality	82	22	90	10	100
Physical role	68.3	40.3	100	0	100
Physical pain	75.3	26.6	84	0	100
General health	55	23.9	57	0	100
Vitality	63.5	23	70	0	100
Social Functioning	70.8	29.6	75	0	100
Emotional role	72.5	40	100	0	100
Mental health	65.9	23.2	72	4	100
Summary physical health scale	47.8	10.3	50.7	18.2	62.3
Summary mental health scale	46.3	12.3	50.5	9.7	64.2

Reducing overall costs due to the disease improved the emotional functioning of the patients. Patients who incurred lower overall costs as a result of the disease expressed fewer apprehensions regarding the future. Some of the patients (n=8, 7.6%) were retired, 61 (58.1%) held employment, and 36 (34.3%) were unemployed. Some of the individuals who held employment (n=29, 49.2%) worked for 40 hours or more per week, while 30 (50.8%) worked for less than 40 hours per week. Public insurance accounted for the plurality of patients (n=95, 90.2%), with insurance covering 103 (98.1%) patients. The monthly household income of 49 (46.6%) patients was equivalent to or less than 1000 euros; 15 patients (14.3%) had an income between 1001 euros and 1500 euros, and 41 patients (39.1%) had an income exceeding 1501 euros after taxes. Over the past six months, patients experienced an average of 6.9 days of work absence, while family members experienced an average of 2.7 days.

During the previous semester, the disease cost an average of 767 euros. The lowest cost was €0, while the greatest cost was €1007. The ranking of cost origins includes items such as medicines, physical therapies, transportation, absence from work, special diets, medical visits, psychologist visits, other expenses, hospital admission, home conversion, care services, nurse visits, and social worker visits. The health system in Greece undertakes a part of the cost as a person insured under the Greek health system pays only part of a contribution to cover certain medical expenses.

In the previous six months, hospital admissions accounted for a range of 12.4%, special diets or nutritional supplements accounted for 29.5%, and various disease-related expenses accounted for 17.1%. Bivariate analysis revealed a statistical relationship at the 0.20 level (p<0.20) between six independent variables and physical health. For this reason, multivariable linear regression was applied, the results of which are presented in Table 3.

**Table 3:** Multivariable linear regression with physical health as the dependent variable.

Independent variable	Coefficient b	95% Interval Confidence for b	p-value
Males versus females	5.1	1.5 to 8.6	0.006
Employees	8.5	5.1 to 11.9	<0.001
Medication	6.7	3.1 to 10.3	<0.001

According to the results of the multivariate linear regression, male employees had better physical health, whereas patients receiving the medication had better physical health. The bivariate analysis revealed that patients with lower total costs due to disease had better physical health, but the multivariate analysis did not maintain this relationship.

At the 0.20 level, bivariate analysis also demonstrated a statistical correlation between physical functioning and eight independent variables (p<0.20). This led to the implementation of multivariable linear regression, with Table 3 summarising the results. The multivariate linear regression analysis demonstrated that patients with higher monthly family income, men, and those on medication exhibited superior functional capacity and physical functioning (Table 4).

**Table 4:** Multivariate linear regression with physical functioning as dependent variable

Independent variable	Coefficient b	95% Interval Confidence for b	p-value
Males versus females	9.4	1.3 to 17.4	0.023
Employees	15.7	7.8 to 23.6	<0.001
Medication	9.1	0.9 to 17.2	0.030

Moreover, a statistical relationship was identified at a level of 0.20 (p<0.20) between three independent variables and social role. This is the reason we implemented multivariate linear regression, and the outcomes are summarized in Table 5. Table 5 shows that employees and patients with lower total costs due to the disease had a more favorable social role, as indicated by the multivariate linear regression.

**Table 5:** Multivariate linear regression with social role as the dependent variable

Independent variable	Coefficient b	95% Interval Confidence for b	p-value
Employees	14.1	3.1 to 25.1	0.013
Total costs due to the disease	-0.008	-0.013 to -0.003	0.004

It is also revealed that there is a statistical relationship between employment and social life at the 0.20 level (p<0.20). For this reason, multivariate linear regression was applied, the results of which are presented in Table 6. The multivariate linear regression revealed that workers had a better social life, and patients with lower total costs due to the disease had a better social life (Table 6).

**Table 6:** Multivariate linear regression with social life as dependent variable

Independent variable	Coefficient b	95% Interval Confidence for b	p-value
Employees	18.1	6.9 to 29.3	0.002
Total costs due to the disease	-0.006	-0.011 to -0.001	0.035

In addition, the multivariate linear regression demonstrated that patients with lower total costs and workers had superior treatment quality of life, which accounted for 9.1% of the treatment variability (Table 7).

**Table 7:** Multivariable linear regression with treatment as the dependent variable

Independent variable	Coefficient b	95% Interval Confidence for b	p-value
Employees	11.1	0.4 to 21.6	0.042
Total costs due to the disease	-0.008	-0.013 to -0.003	0.003

Additionally, it appeared that the residents of Attica had superior emotional functioning. Reducing overall costs due to the disease improved the emotional functioning of the patients. Patients who incurred lower overall costs as a result of the disease expressed fewer apprehensions regarding the future.

## Discussion

The research revealed that cystic fibrosis has a substantial influence on the quality of life of patients, irrespective of their age. The study also revealed that the disease's average total annual cost is 767 euros. Furthermore, 17.1% of patients incurred additional expenses related to the social and physical consequences of the disease, rather than solely for treatment administration. Indeed, patients experienced below-average quality of life in terms of both their mental and physical well-being.

This study is the first that tries to assess the influence of CF on CFQoL and the overall societal cost of a population of patients. Previous studies have primarily focused on the immediate costs associated with healthcare or on particular populations from randomised controlled trials or comprehensive database claims to estimate their projected expenses. The chosen methodology enabled the inclusion of expenses associated with informal care, which represent only a small portion of the total cost of the disease [18].

Other global studies have also examined the costs associated with CF. However, most of these studies have focused exclusively on the direct healthcare costs, while only a few have taken into account the informal and indirect costs. The direct healthcare costs exhibit significant variation across countries, with Australia documenting a cost of US$15,571 in 2003 and 2005. Comparing these findings with those of this investigation is challenging due to differences in the study populations (clinical trial patients, hospitalized patients, or individuals with private insurance) and variations in the cost factors used to calculate expenses. The Australian study revealed that hospitalization and medications accounted for the majority of costs, whereas the American study revealed that drug expenses accounted for a significantly larger proportion of costs. It is not surprising that the cost of medicines in the United States is undeniably higher than in Europe [19,20].

The German study, which reported the highest costs, found that drug expenses made up 81% of the total expenses. However, it is important to note that the variation in patient characteristics can account for this difference. Out of the studies mentioned earlier, only two also investigated the indirect expenses associated with CF. Both studies reported significantly lower costs: €2,491 in Germany [25] and $1,180 in the USA [26]. The present study's results were more consistent with these findings. However, neither included expenses related to early retirement.

Both the present study and the study conducted by Debska and Mazurek found that patients experienced a diminished quality of life as a result of their apprehension about engaging in work or study [27]. The current study found a notable correlation between patients' occupational status and their quality of life in the area of work or study, based on sociodemographic factors. Individuals who were unemployed experienced a diminished quality of life in this particular area. The progress made in the treatment of cystic fibrosis has greatly extended the average lifespan of patients, enabling them to lead healthier lives and actively engage in their professional careers. Approximations indicate that over half of European patients who are unemployed and not actively seeking employment (18-30%) attribute their work cessation or inactivity to cystic fibrosis. Employed individuals had a more favorable perception of their profession in comparison to individuals with a higher monthly household income. This appears to be unrelated to the specific clinical condition and the treatment employed [27]. The German study, which reported the highest costs, found that drug expenses made up 81% of the total costs. However, it is important to note that the variation in patient characteristics can account for this difference. Out of the studies mentioned earlier, only two also analyzed the indirect expenses associated with CF [25,26]. The present study's results were more consistent with these findings. However, neither included expenses related to early retirement.

Limitations

Although the current research provides valuable insights into the correlation between the cost of living and patients with CF, it is crucial to recognize various limitations. Furthermore, the study focuses primarily on patients from a single hospital, with whom they maintain ongoing monitoring. The sample size of 105 participants, while providing a substantial response rate, may still limit the ability to generalize the results. Subsequent investigations should strive to rectify these constraints and yield more universally applicable findings.

## Conclusions

It appears that implementing a plan regarding treatment that minimizes the cost of living in these patients, can potentially enhance overall life satisfaction. Undoubtedly, patients with lower overall costs due to disease had better quality of life. Working conditions, salary and job satisfaction may affect the cost of living of patients with CF. Therefore, there is a requirement for ongoing surveillance of the quality of life of patients with cystic fibrosis, in conjunction with the implementation of novel or enhanced treatment approaches.
